# High Frequency and Diversity of Antimicrobial Activities Produced by Nasal *Staphylococcus* Strains against Bacterial Competitors

**DOI:** 10.1371/journal.ppat.1005812

**Published:** 2016-08-04

**Authors:** Daniela Janek, Alexander Zipperer, Andreas Kulik, Bernhard Krismer, Andreas Peschel

**Affiliations:** 1 Interfaculty Institute of Microbiology and Infection Medicine, Infection Biology, Eberhard-Karls-University Tübingen, Tübingen, Germany; 2 German Center for Infection Research, Partner site Tübingen, Tübingen, Germany; 3 Interfaculty Institute of Microbiology and Infection Medicine, Microbiology/Biotechnology, Eberhard-Karls-University Tübingen, Tübingen, Germany; National Jewish Health, UNITED STATES

## Abstract

The human nasal microbiota is highly variable and dynamic often enclosing major pathogens such as *Staphylococcus aureus*. The potential roles of bacteriocins or other mechanisms allowing certain bacterial clones to prevail in this nutrient-poor habitat have hardly been studied. Of 89 nasal *Staphylococcus* isolates, unexpectedly, the vast majority (84%) was found to produce antimicrobial substances in particular under habitat-specific stress conditions, such as iron limitation or exposure to hydrogen peroxide. Activity spectra were generally narrow but highly variable with activities against certain nasal members of the *Actinobacteria*, *Proteobacteria*, *Firmicutes*, or several groups of bacteria. *Staphylococcus* species and many other *Firmicutes* were insusceptible to most of the compounds. A representative bacteriocin was identified as a nukacin-related peptide whose inactivation reduced the capacity of the producer *Staphylococcus epidermidis* IVK45 to limit growth of other nasal bacteria. Of note, the bacteriocin genes were found on mobile genetic elements exhibiting signs of extensive horizontal gene transfer and rearrangements. Thus, continuously evolving bacteriocins appear to govern bacterial competition in the human nose and specific bacteriocins may become important agents for eradication of notorious opportunistic pathogens from human microbiota.

## Author Summary

The complex and dynamic microbial communities of human body surfaces are of utmost importance for human body functions in health and diseases. Human microbiomes contribute to metabolic processes, instruct the immune system, and often include antibiotic-resistant pathogens, responsible for the majority of severe bacterial infections. It is generally accepted that microbiota composition is strongly affected by mechanisms of microbial interference, but how specific bacteria may achieve fitness benefits and outcompete other microbes has remained largely unknown. We demonstrate that production of antimicrobial bacteriocins is not an occasional trait but a dominant and highly variable strategy among human nasal bacteria for limiting the growth of competing microbes. We found that more than 80% of nasal *Staphylococcus* isolates produce bacteriocins with highly diverse activity spectra, in particular under habitat-specific stress conditions such as iron limitation and exposure to hydrogen peroxide. Inactivation of a representative bacteriocin diminished the producer’s competitive capability indicating that bacteriocins may be a major driving force for the dynamics of microbiomes in nutrient-poor habitats such as the human nose. The identification of bacteriocin genes on mobile genetic elements with composite structure suggests that they are subject to highly dynamic co-evolutionary processes.

## Introduction

The microbiomes of human body surfaces contribute to health and wellbeing in multiple ways [1, 2]. At the same time, they represent major reservoirs for many human bacterial pathogens such as *Staphylococcus aureus* and members of the *Enterococcaceae*, *Streptococcaceae*, and *Enterobacteriaceae*. The composition of microbiota is highly variable between different human individuals and it changes over time [3]. Environmentally exposed microbiomes such as those of the human skin and airways are much more variable than those of the gut [4]. Changing environmental stressors, climate, personal hygiene, and contact to other persons contribute to the dynamic alteration of skin and airway microbiota [3, 5–7].

The success of individual bacterial strains in competition with other microbes is governed by multiple types of interaction [8–11]. The underlying mechanisms are complex and have been hardly explored. They may include different capabilities to bind to a limited number of host attachment sites, to take up and metabolize scant nutrients, or to produce antibacterial substances, referred to as bacteriocins, that inhibit competitors. Recent studies on the availability of nutrients in the human nose indicated that this habitat is extremely poor [12] compared e.g. with the gastrointestinal tract [13], which is constantly exposed to ingested food. Accordingly, the nasal microbiome is less dense and less diverse than the intestinal microbiome [5, 14], and it is tempting to assume that bacterial strains that colonize the nose have efficient means to compete with other bacteria. [3, 5].

Most of the *Staphylococcus* species found in the nose are either exclusive commensals (*Staphylococcus hominis*) or cause infections in hospitalized patients with indwelling devices (*Staphylococcus epidermidis*, *Staphylococcus warneri*, *Staphylococcus lugdunensis Staphylococcus capitis*, and others). In contrast, *Staphylococcus aureus* is not only an opportunistic but a highly aggressive pathogen that is able to infect also healthy humans. *S*. *aureus* is one of the most frequent causes of skin and soft-tissue infections as well as blood stream infections often resulting in endocarditis or sepsis [15, 16]. Only one third of the human population is permanently colonized by *S*. *aureus* and carriers are at a higher risk of invasive *S*. *aureus* infections [17, 18]. The preferred *S*. *aureus* niche is the anterior nares and nasal eradication of *S*. *aureus* with the antibiotic mupirocin is a valuable strategy to reduce the risk of endogenous infections in immunocompromised patients [19, 20]. Why only certain persons are colonized and why this trait is stable over time has remained unknown. While host genetic factors have been thought to play a role in the carrier status [21], a recent twin cohort study suggests that other than genetic factors may be more important [22]. These may include mechanisms of microbial interference in the nose, but the identity and frequency of such processes have not been elucidated.

Bacteria found in the human nose or in other habitats have been occasionally reported to produce ribosomally synthesized antimicrobial peptides referred to as bacteriocins [23–25]. Bacteriocin prepeptides are often modified e.g. by formation of thioether rings in the case of lantibiotics, and producing strains have immunity genes providing resistance specifically to the cognate bacteriocin but not to other antimicrobial compounds [26]. Most of the known bacteriocins have rather narrow activity spectra, often against closely related bacteria, which may reflect their specific ecological roles but remains an obstacle for their use as antimicrobial agents [27]. Bacteriocin genes are usually encoded on mobile genetic elements (MGEs) such as plasmids and only rarely found in bacterial core genomes [23, 24, 28]. All these findings point to diverse and important roles of bacteriocins in bacterial interference and microbiota composition, but systematic analyses of bacteriocin abundance, diversity, and activity spectra in a given microbial niche have not been conducted so far.

In this paper, to examine the ability of nasal *Staphylococcus* strains to produce antimicrobial substances and substances' activity spectra, we tested 89 human nasal *Staphylococcus* isolates against a selection of nasal commensal bacteria. We further characterized the structure and capacity of a newly identified *S*. *epidermidis* bacteriocin with a specific activity spectrum, and evaluated its impact on other nasal commensals.

## Results

### A large number of nasal *Staphylococcus* strains produce antimicrobial substances, which differ largely in activity spectra

**Fig 1:**
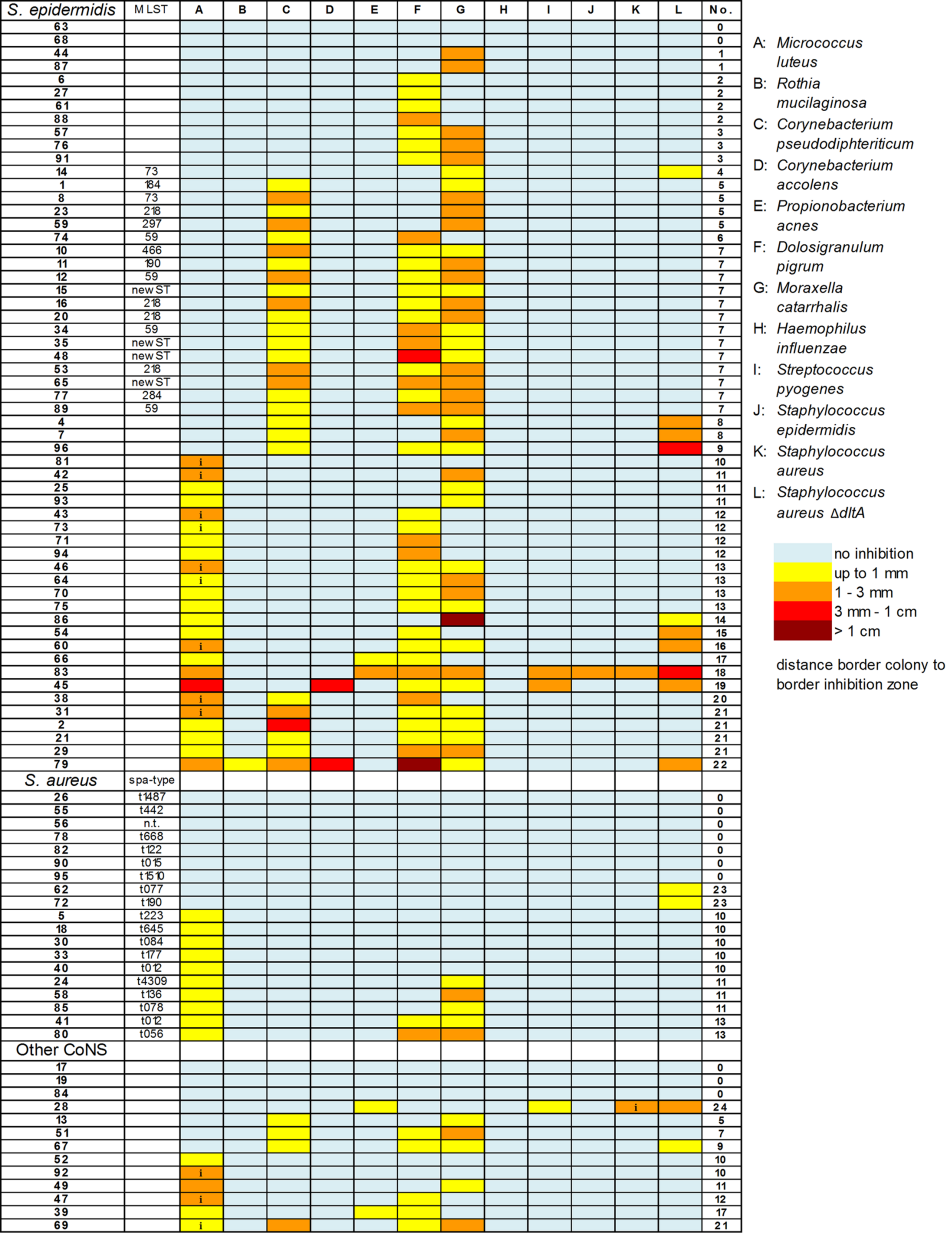
Frequency and activity spectra of antimicrobial substances produced by nasal *Staphylococcus* isolates. Pattern and intensity of test strain inhibition by nasal *Staphylococcus* isolates is shown as a heat map. They are ordered hierarchically by activity patterns according to the number and identity of inhibited strains (indicated by numbers in the last column) against *Actinobacteria* (A-E); *Proteobacteria* (F-G); *Firmicutes* (H-J). (i) indicates inducible bacteriocin production, which was only visible under iron-limitation stress. MLST types are given for 19 *S*. *epidermidis* strains and *spa*-types are indicated for all *S*. *aureus* isolates. (n.t.; non-typeable).

To determine the frequency and diversity of bacteriocin production by nasal *Staphylococcus* species, and if their bacteriocins preferentially inhibit other nasal bacteria, we analyzed 89 *Staphylococcus* isolates (IVK strains) recovered from the noses of 37 healthy human volunteers. These strains belonged to six different species (*S*. *epidermidis*, *S*. *aureus*, *S*. *hominis*, *S*. *lugdunensis*, *S*. *warneri* and *S*. *capitis*). Whereas these species have been reported in the human nose in several independent studies [29–31], the collected strains may represent a random and not necessarily representative panel of *Staphylococcus* strains form the nasal microbiota. Eleven bacterial species, frequently found in human nasal microbiomes [5], were used as test strains for the identification of inhibition zones around IVK colonies. They comprised representatives from the *Actinobacteria* families *Micrococcaceae* (*Micrococcus luteus*, *Rothia mucilaginosa)*, *Corynebacteriaceae (Corynebacterium pseudodiphteriticum*, *Corynebacterium accolens)*, and *Propionibacteriaceae (Propionibacterium acnes)*, *Firmicutes* families *Staphylococcaceae* (*S*. *aureus*, *S*. *epidermidis)*, *Carnobacteriaceae (Dolosigranulum pigrum)*, *Streptococcaceae (Streptococcus pyogenes*), and *Proteobacteria* families *Moraxellaceae* (*Moraxella catarrhalis)* and *Pasteurellaceae (Haemophilus influenzae*). Notably, production of antimicrobial compounds was a common and highly variable phenotype among the *Staphylococcus* strains tested (Figs 1 and S1) with 84% of them inhibiting at least one of the test strains. In total, 77 strains exhibited antimicrobial activity, but the various *Staphylococcus* isolates differed largely in the strains targeted by their antimicrobial substances. While the majority of *S*. *epidermidis* inhibited *D*. *pigrum* and *M*. *catarrhalis*, only some *S*. *aureus* were found to target these species. Conversely, the majority of *S*. *aureus* but only some *S*. *epidermidis* inhibited *M*. *luteus*. Whereas none of the *S*. *aureus* strains was able to inhibit *C*. *pseudodiphteriticum*, almost half of all *S*. *epidermidis* isolates showed this capacity. *S*. *epidermidis* was by far the most frequent bacteriocin-producing *Staphylococcus* species (96%), whereas only 69% of other CoNS and 52% *of S*. *aureus* isolates showed inhibitory activity (Table 1).

**Table 1:** Frequency of antimicrobial activitiy in nasal *Staphylococcus* isolates.

	Antimicrobial activity (in % of strains) against:				
	*M*. *luteus*	*C*. *pseudodiphtheriticum*	*D*. *pigrum*	*M*. *catarrhalis*	Any of the four strains
***S*. *epidermidis***	**24/57 (42.1)**	**27/57 (47.4)**	**41/57 (71.9)**	**42/57 (73.7)**	**55/57 (96)**
**Other CoNS**	**6/13 (46.2)**	**4/13 (30.8)**	**5/13 (38.5)**	**5/13 (38.5)**	**9/13 (69)**
***S*. *aureus***	**10/19 (52.6)**	**0/19 (0)**	**2/19 (10.5)**	**5/19 (26.3)**	**10/19 (52)**

The inhibition patterns of antimicrobial compounds from our bacteriocin-producing IVK strain panel were highly diverse and most had a rather narrow spectrum that covered one or two (49.4%) or three to four (46.8%) of the test strains. According to the panel of inhibited strains 24 different inhibition patterns were defined. Even though it cannot be excluded that some strains may differ in the amounts of the produced compounds or may produce more than one antimicrobial, these findings suggest that there is a remarkable variety of antimicrobials produced by nasal *Staphylococcus* strains. In order to elucidate if specific inhibitory patterns are associated with particular clonal lineages, the clonal identities of 19 *S*. *epidermidis* isolates with inhibitory patterns no. 4 to 7 and of all 19 *S*. *aureus* isolates were analyzed by multi-locus sequence typing (MLST) and spa typing, respectively. However, with only a few exceptions, the inhibitory patterns and the clonal identities did not correlate, suggesting that bacteriocin production is a very variable trait that may often depend on acquisition of MGEs (Fig 1, S1 and S2 Tables).

*Staphylococcus* strains encounter a wide range of different bacterial species in the nasal microbiota, and it is largely unclear, which bacteria may be major competitors and whose inhibition by a particular bacteriocin may provide a significant fitness benefit for the producer. Among the 11 test strains *M*. *luteus*, *C*. *pseudodiphteriticum*, *D*. *pigrum*, and *M*. *catarrhalis* were most frequently inhibited by bacteriocins from the majority of *S*. *epidermidis* and some of the *S*. *aureus* or other CoNS strains (Fig 1), suggesting that interfering with growth of these species may provide particular fitness benefits. Only sporadic activities were found against *C*. *accolens*, *P*. *acnes*, *R*. *mucilaginosa*, *S*. *pyogens*, or other *Staphylococcus* strains from the species *S*. *epidermidis* and *S*. *aureus* (Fig 1), but none of the IVK strains was able to inhibit *H*. *influenzae*. Several of the detected bacteriocins inhibited preferentially *Proteobacteria* (*M*. *catharrhalis*) or *Actinobacteria* species (e.g. *M*. *luteus* or C. *pseudodiphteriticum*) but many combined activities against certain *Proteobacteria* and *Actinobacteria* species. Inhibition of *Firmicutes* was generally rare except for *D*. *pigrum*, which was susceptible to many of the detected bacteriocins from *S*. *epidermidis* and other CoNS. Only a small minority of the IVKs (three strains) exhibited broad-spectrum antimicrobial activity against the majority of test strains. Such molecules may represent interesting candidates for new antibiotics.

While only one of the IVK strains inhibited wild-type *S*. *aureus* under standard conditions, this number increased to fourteen when an isogenic *S*. *aureus* mutant, lacking the d-alanine modification of teichoic acids, was used (Δ*dltA*; Fig 1, column L). Teichoic acid alanylation modifies the net charge of the cell envelope and leads to resistance to many cationic antimicrobials [32]. Thus, many bacteriocins can act against *S*. *aureus*, but these bacteria appear to be well protected by their intrinsic antimicrobial peptide resistance mechanisms.

### Many bacteriocins are induced in *Staphylococcus* isolates by colonization-related stress conditions

*Staphylococcus* species have quite different life styles and habitats [15, 18, 33] raising the possibility that they may regulate bacteriocin production and express bacteriocin genes only under colonization-related conditions. In order to simulate stress conditions encountered in the human nose, we analyzed antimicrobial activities of the 89 IVK strains under conditions of iron limitation and in the presence of H_2_O_2_. Bacteria have been found to strongly up-regulate genes of iron acquisition [12, 34] and to be exposed to H_2_O_2_ released by *Streptococcus* strains or by phagocytes in the human nose [35–37]. *M*. *luteus* and *S*. *aureus* were chosen as representative test strains that were inhibited only by a minority or by almost no IVK strains, respectively (Fig 1), and because they grew well on test agar plates with iron limitation or H_2_O_2_.

**Fig 2:**
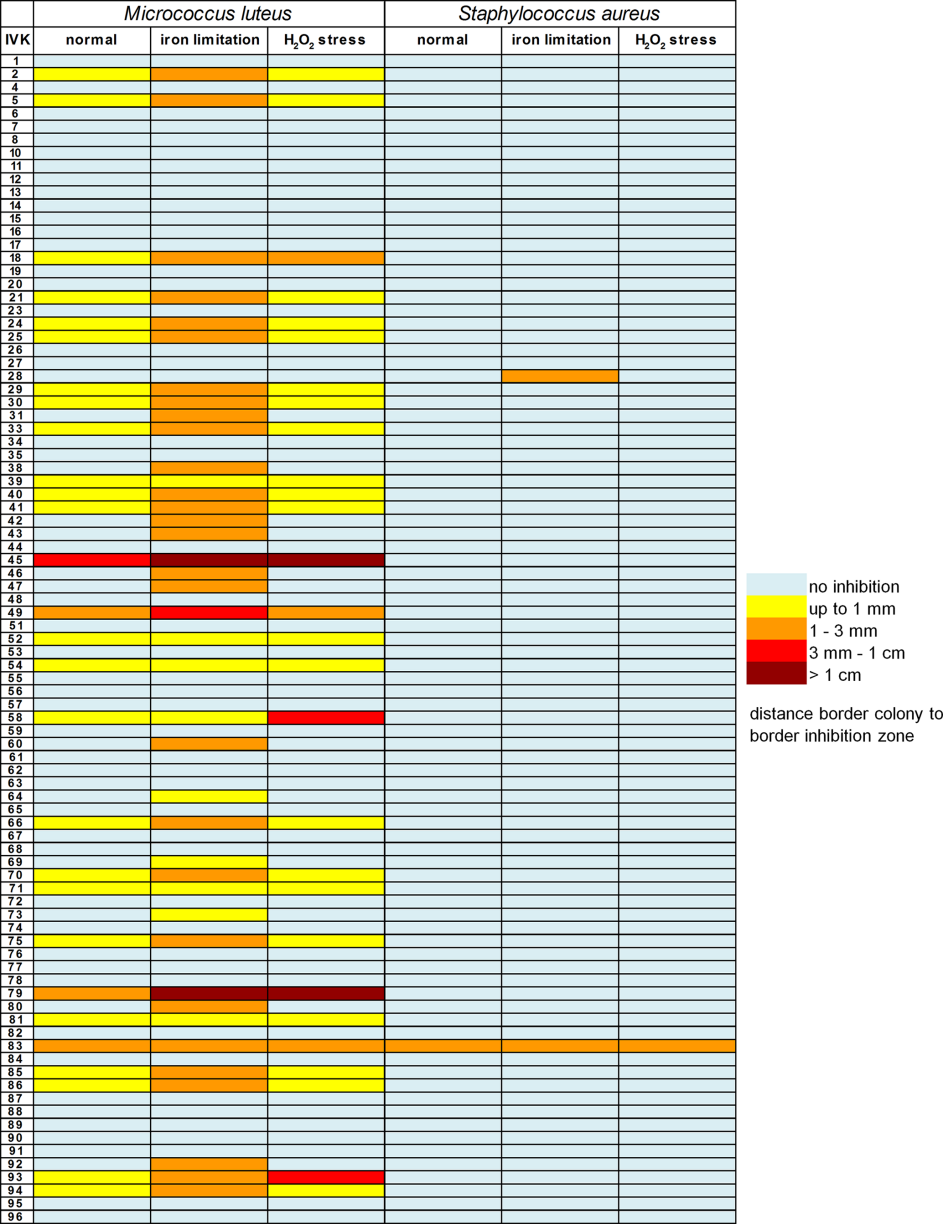
Bacteriocin induction by iron limitation or H_2_O_2_. Intensity of inhibitory activities of IVK strains 1–96 against *M*. *luteus* or *S*. *aureus* without stressors (normal) or in the presence of 2,2’-bipyridine (iron limitation) or H_2_O_2_ (H_2_O_2_ stress).

Addition of the iron chelator 2,2’-bipyridine at a final concentration of 200 μM to test agar plates increased IVK strains’ antimicrobial activity against *M*. *luteus* by varying degrees; however, some IVK strains did not inhibit *M*. *luteus*, irrespective of bipyridine addition (Figs 2 and S2). In contrast, the addition of bipyridine did not substantially change IVK strains’ antimicrobial activity against *S*. *aureus*, except for strain IVK28. The vast majority (82.5%) of the activities against *M*. *luteus* or *S*. *aureus*, were only detectable (32.5%) or substantially increased (52.5%) under conditions of iron limitation (Fig 2).

H_2_O_*2*_ did not elicit bacteriocin production against *M*. *luteus* or *S*. *aureus* in any of the non-producers but 18% of the activities active against *M*. *luteus* were increased in the presence of H_2_O_2_ (Figs 2 and S2). Taken together, these data show that many nasal *Staphylococcus* strains regulate bacteriocin production and release antimicrobial substances only under conditions resembling their nasal habitat.

### *S*. *epidermidis* strain IVK45 produces a nukacin-related lantibiotic

The highly diverse antimicrobial activities among nasal *Staphylococcus* isolates and the fact that the type of inhibition pattern did not correlate with clonal identity suggests that different bacteriocin genes may have been repeatedly introduced and exchanged by *Staphylococcus* strains. *S*. *epidermidis* strain IVK45 was chosen to elucidate the molecular basis of its bacteriocin production, because this strain showed strong antimicrobial activity against several unrelated bacteria (*M*. *luteus*, *C*. *accolens*, *S*. *pyogenes*, *D*. *pigrum*, *M*. *catarrhalis;* Fig 1) that was inducible by H_2_O_2_ and iron limitation (Fig 2). The genes responsible for bacteriocin production were identified by sequencing of a transposon mutant lacking antimicrobial activity and found on a plasmid, subsequently named pIVK45, of 21.840 nucleotides (accession number KP702950).

**Fig 3:**
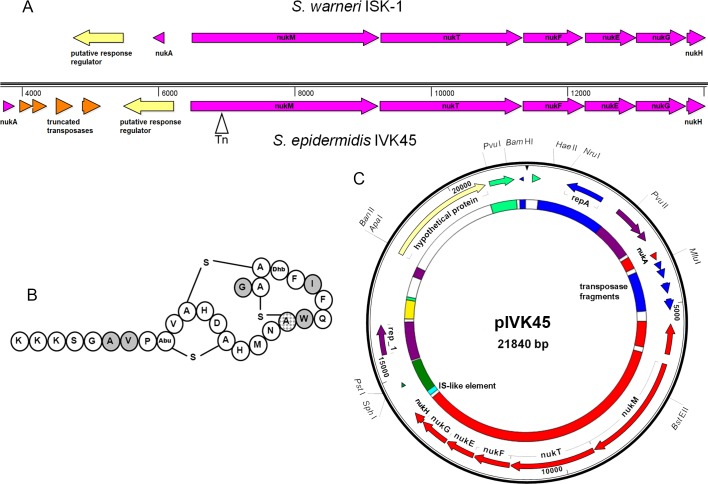
Nukacin IVK 45 operon, predicted peptide structure and composition of plasmid pIVK45. A: comparison of the operon structures from *S*. *warneri* ISK-1 (top) and *S*. *epidermidis* IVK45 (bottom), Tn: insertion site of the transposon. B: Predicted structure of nukacin IVK45. Amino acid positions of nukacin IVK45, which are different in corresponding peptides from *S*. *warneri* ISK-1 and *S*. *hominis* KQU-131 are shown in grey. The additional different amino acid in *S*. *hominis* KQU-131 is shown in a grey pattern; A-S-A, lanthionine (thioether bridge between cysteine and serine); Abu-S-A, 3-methyllanthionine (thioether bridge between cysteine and threonine); Abu, aminobutyrate (threonine within the methyllanthionine ring); Dhb, dehydrobutyrine (dehydrated threonine). C: Intact genes or fragments for transposases, recombinases, IS- and IS-like elements indicate multiple recombination events in the genesis of pIVK45. Outer ring of plasmid: identified genes are indicated by arrows. Inner ring: The color of the various segments indicates their most likely species origin (analyzed by BLAST). Red: *S*. *warneri*, blue: *S*. *aureus*, light green: *S*. *epidermidis*, dark green: *S*. *aureus* and *S*. *epidermidis*, yellow: *S*. *lugdunensis*, lilac: many different *Staphylococcus* species, light blue: IS-like element. White segments show unique DNA fragments with no homologies in available databases.

The bacteriocin gene cluster exhibited similarity to genes required for biosynthesis of a ribosomally synthesized lanthionine-containing bacteriocin (lantibiotic). The highest similarity was found with genes previously described in *S*. *warneri* ISK-1 [38] and *S*. *hominis* KQU-131 [39] to be required for biosynthesis of the lantibiotic nukacin (Fig 3A). The mature bacteriocin of *S*. *epidermidis* IVK45 was predicted to consist of 27 amino acids including a dehydrobutyrine, a 3-methyllanthionine, and two lanthionine residues, originating from threonine, cysteine, and serine, respectively (Figs 3B and S3). It differs from nukacin ISK-1 and KQU-131 by five and six amino acids, respectively, in the mature peptide and another five amino acids in the leader peptide (S3 Fig).

To further confirm its identity, the compound named nukacin IVK45 was purified from culture filtrates by a combination of adsorption, cation exchange, and size exclusion chromatography. The resulting active fraction contained only one notable peak in RP-HPLC chromatograms that was absent from the corresponding preparation of the nukacin-deficient mutant (S4A and S4B Fig). Mass spectrum (MS) analysis confirmed that the peak corresponds to nukacin IVK45 (S4C Fig) and matches the calculated mass of 2,940 Da. Hence, this is the first report of a nukacin-like lantibiotic in *S*. *epidermidis*.

### Nukacin IVK45 is encoded on a composite plasmid whose sequence suggests a history of extensive horizontal gene transfer and rearrangements

The precursor sequence of nukacin IVK45 does not only align with those of nukacin ISK-1 and KQU-131 from *S*. *warneri* and *S*. *hominis*, respectively, but also with several hypothetical peptides from other bacterial genomes (S3 Fig). A peptide from *Streptococcus agalactiae* was particularly similar to nukacin IVK45, whereas peptides from other *Firmicutes* from the genera *Streptococcus*, *Lactococcus*, or the *Actinobacteria* species *Kocuria* were less related. Thus, lantibiotic genes appear to be exchanged even between bacteria from different orders and phyla.

The nukacin IVK45 genes were located on an MGE with obvious composite structure. The operon was most similar to that of *S*. *warneri* ISK-1, but the location and orientation of the precursor peptide gene *nukA* was different (Fig 3A). The other parts of pIVK45 including the origin of replication, open reading frames encoding replication and hypothetical proteins have highly similar counterparts in plasmids or other MGEs from *S*. *aureus*, *S*. *epidermidis*, and *S*. *lugdunensis* (Fig 3C) underscoring a common DNA exchange between several *Staphylococcus* strains and species.

### Nukacin IVK45 increases fitness of *S*. *epidermidis* in competition with microbial competitors

**Fig 4:**
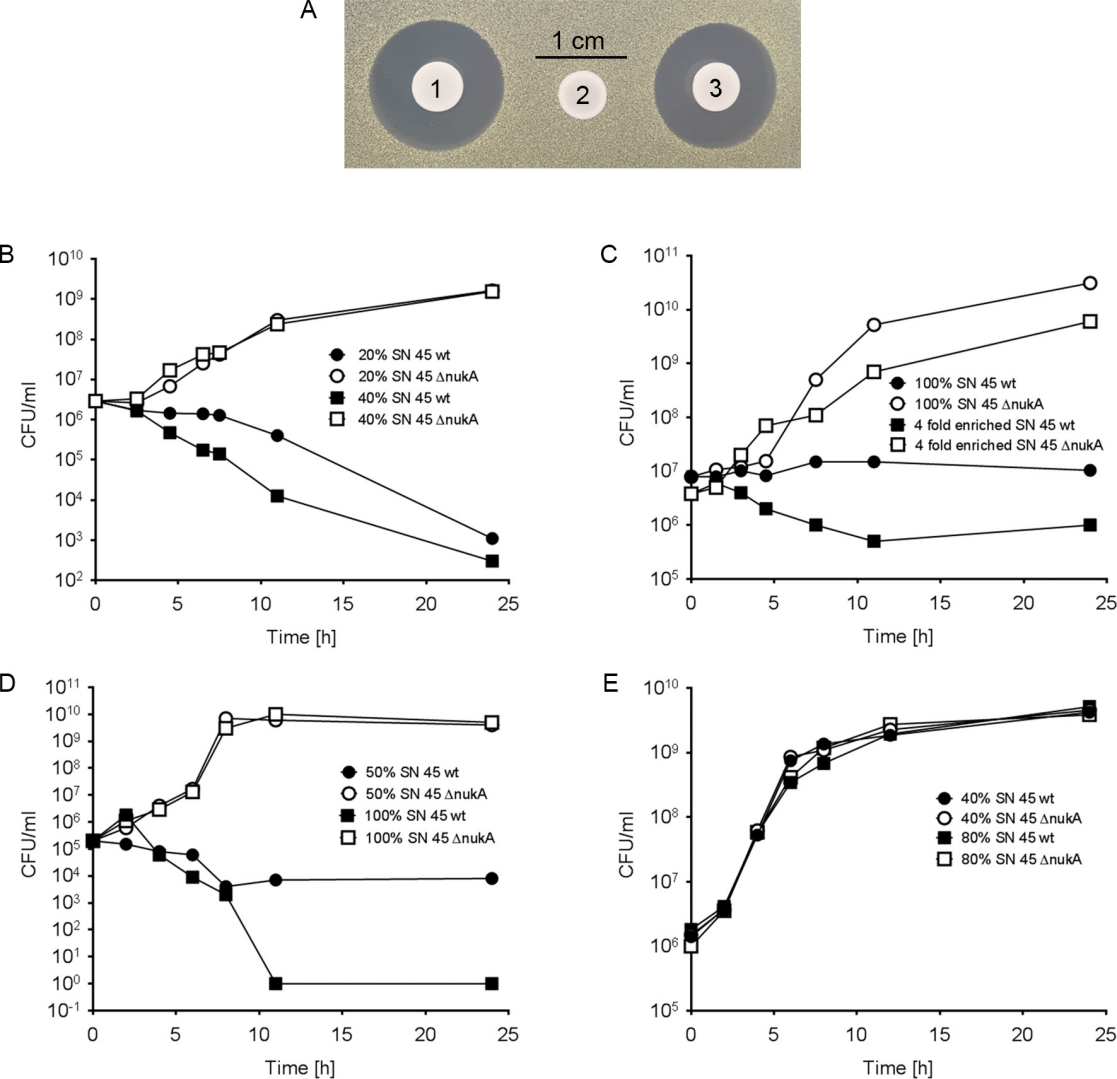
Antibacterial activity of IVK45 wild type compared to nukacin deletion mutant and complemented mutant. A: IVK 45 wild type (1); nukacin-deficient mutant Δ*nukA* (2); and complemented mutant (3) on *M*. *luteus* lawns. B: *M*. *luteus* cultures supplemented with 20% and 40% supernatant (SN) of IVK 45 wild type and nukacin-deficient mutant; C: *M*. *catarrhalis* cultures in spent medium or supplemented with 4-fold concentrated activity of IVK 45 wild type and nukacin-deficient mutant; D: *C*. *accolens* cultures in spent medium or supplemented with 50% supernatant of IVK 45 wild type and nukacin-deficient mutant; E: Nukacin insensitive *S*. *aureus* Newman cultures supplemented with 40% and 80% supernatant of IVK 45 wild type and nukacin-deficient mutant as negative control.

In order to study how the production or absence of a bacteriocin may impact on the competitive capacities of nasal bacteria, a defined *S*. *epidermidis* IVK45 mutant, lacking the *nukA* gene, was constructed, which lacked antimicrobial activity as expected (Fig 4A). The plasmid pRB474-nukA was used for complementing the mutant with a wild-type copy of *nukA* expressed from a constitutive promoter. The complemented strain resumed production of antimicrobial activity thereby confirming that the *nuk* genes and their product are responsible for the bacteriocin activity of IVK45.

**Fig 5:**
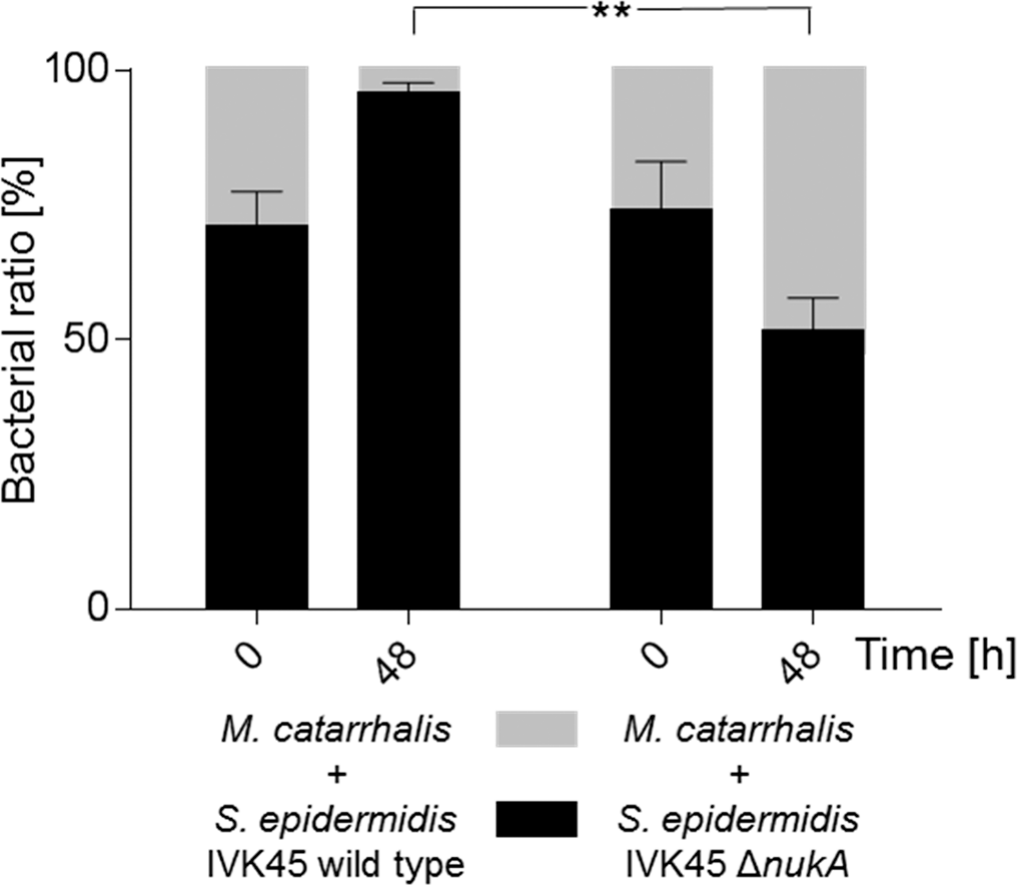
Co-cultivation of *M*. *catarrhalis* and *S*. *epidermidis* IVK45 strains. *S*. *epidermidis* IVK45 wild type and mutant IVK45 Δ*nukA* (black) were inoculated at ratios of 3:1 with *M*. *catarrhalis* (grey) on solid agar. *M*. *catarrhalis* is overgrown by the nukacin-producing IVK45 wild type after 48 hours. In contrast, the numbers of the nukacin-deficient mutant IVK45 Δ*nukA* and *M*. *catarrhalis* shift towards a ratio of 1:1 after 48 hours. Significant differences between the IVK45 wild type and mutant Δ*nukA* ratios after 48 hours were analyzed by two tailed paired t-test (** *P* < 0.005).

To further investigate how nukacin IVK45 may affect the growth of other nasal bacteria, *M*. *luteus*, *C*. *accolens*, and *M*. *catarrhalis* were cultivated in medium supplemented with culture filtrate of IVK45 wild type or the nukacin-deficient mutant (Fig 4B to 4D). The CFU per ml of *M*. *luteus* and *C*. *accolens* decreased almost 10,000-fold within 24 hours in cultures containing culture filtrates of IVK 45 wild type, while culture filtrates of the isogenic *nukA* mutant did not inhibit growth of the test strains. Increasing amounts of culture filtrate of IVK45 wild type showed dose-dependent inhibition of the test strains. Culture filtrate of IVK45 but not of the *nukA* mutant prevented also growth of *M*. *catarrhalis* but did not lead to a major reduction of viable cells even if nukacin IVK45 was strongly concentrated. Growth of nukacin-insensitive *S*. *aureus* Newman, which was included as a negative control, was not affected by IVK45 or IVK45 Δ*nukA* culture filtrate (Fig 4E). Co-cultivation of IVK45 wild type or ∆*nukA* with *M*. *catarrhalis* on agar plates demonstrated that nukacin IVK45 allows *S*. *epidermidis* to outcompete *M*. *catarrhalis* (Fig 5). Thus, nukacin IVK45 is a bactericidal or bacteriostatic bacteriocin, depending on the target strain, with a strong capacity to interfere with growth of susceptible nasal bacteria.

## Discussion

Bacteriocins have been described in many bacterial species, but their ecological significance has remained unclear [27, 40]. Specifically, we are lacking insights into the frequency, diversity, regulation, and activity spectra of bacterial antimicrobial molecules in a given microbial niche. It is obvious that bacteriocins can augment bacterial fitness when producing strains reside in a complex microbiota such as those of human body surfaces. The poor availability of nutrients in the nose [12], compared to other human body surfaces, such as those of the gastrointestinal tract [13], may cause a particularly high competitive pressure and may be a reason why we found an unexpectedly high frequency and diversity of bacteriocins in nasal bacteria.

The effectiveness of a particular bacteriocin should depend on its capacity to restrict growth of most critical competitors of the producing strain. It is interesting to note that the investigated bacteriocins from *Staphylococcus* strains varied largely in activity spectra suggesting that this group of bacteria does not have a preferred competitor but may need to combat quite different bacteria in different hosts. The differences in activity spectra may reflect the enormous variability in composition of individual nasal microbial communities, which can be dominated by different bacterial genera such as *Moraxella*, *Corynebacterium*, *Propionibacterium*, or *Staphylococcus* [5, 29, 41–43] depending on the individual host. On the other hand, the activity spectra of bacteriocins produced in a particular nose may as well contribute to the capacity of a given bacterial species to dominate the microbiota. If this hypothesis holds true, then there should be a chance for developing ‘probiotic’ strains that would support a favorable microbiota composition and could eradicate facultative pathogens such as *S*. *aureus*. To this end it will be crucial to associate microbiota composition and pathogen colonization with bacteriocin diversity in large cohorts of human volunteers in the future.

Individual nasal bacterial strains have been found to affect each other in specific ways. For instance, H_2_O_2_ produced by *Streptococcus* strains, living in close proximity to *Staphylococcus* strains on human body surfaces [43], can induce prophages in *S*. *aureus*, which destroy *Staphylococcus* cells during the propagation process [37]. Our finding that H_2_O_2_ also induces certain bacteriocins from *Staphylococcus* strains suggests that these bacteria can fight back and produce anti-*Streptococcus* compounds. There is evidence that an inverse correlation exists between *Staphylococcaceae* and *Corynebacteriaceae*, and it has been shown that high numbers of *Corynebacterium* species but also of of *S*. *epidermidis* can limit nasal colonization by *S*. *aureus* [43–46]. Our study suggests that this competition is governed at least in part by bacteriocins. Of note, only two of 77 inhibitory activities were found to be active against *S*. *aureus*, supporting the notion that this pathogen has particularly effective resistance mechanisms against antimicrobial peptides encoded by the *dltABCD* and *mprF* genes [47]. In contrast to the wild type, the Δ*dltA* mutant of *S*. *aureus* (Fig 1, panel L) was sensitive to compounds from 14 of the investigated strains including nukacin IVK45.Therefore, it is very likely that such compounds resemble cationic antimicrobial peptides (like nukacin IVK45), against which the Δ*dltA* mutant is most sensitive [32].

*S*. *aureus* nasal colonization depends on expression of several molecules, such as host ligand-binding surface proteins, teichoic acids, capsule, and proteases [18]. *S*. *aureus* is eradicated from the nose of risk patients, but increasing resistance rates against mupirocin, the antibiotic currently used for eradication, demand the development of new decolonization strategies [48]. Identifying and harnessing bacteriocins that act against *S*. *aureus* will be crucial for potential future strategies for selective eradication of *S*. *aureus*. It is interesting to note that nasal bacteria do not only inhibit each other but, in certain instances, appear also to depend on each other and support each other’s growth [45]. Such growth-promoting capacities may be based on release of specific nutrients or on production of siderophores subducted by other bacteria that cannot produce but take up such iron-scavenging molecules. Thus, there are probably several other processes governing the fitness of bacteria in the nasal microbiome in addition to production of or resistance to bacteriocins. Such mechanisms may explain why 16% of the nasal *Staphylococcus* isolates from this study can colonize the nose without producing bacteriocins.

The observed diversity of inhibition patterns of nasal bacteriocins implies that there is a substantial pool of bacteriocin-encoding MGEs that are frequently rearranged and horizontally transferred or exchanged between different nasal strains. In line with this assumption most bacteriocins are encoded on plasmids [23, 24, 28] that can be easily exchanged either by transducing phages or by conjugative elements [49–51]. Such horizontal gene transfer events can be observed across the boundaries of species and genera and are directed e.g. by the capacity of a transducing phage to bind to wall teichoic acids in donor and recipient strains [52]. Transposases and recombinases found on pIVK45 and many other MGEs [53, 54] may contribute to recombination events that continuously create new bacteriocin variants with altered activity spectra. Nukacin IVK45 may be regarded as a paradigm for bacteriocin evolution: It is encoded together with several gene fragments of transposases, recombinases, IS- and IS-like elements on a mosaic plasmid composed of DNA segments probably originating from many different bacterial species in combination with other MGEs and DNA fragments. It differs from the first discovered nukacin ISK-1 from *S*. *warneri* in five amino acid positions in the mature peptide, which may have altered its activity spectrum. Along this line, nukacin ISK-1 has been described to be bacteriostatic for *S*. *aureus* and other Gram-positive and bactericidal for *M*. *luteus* and *Staphylococcus simulans* but inactive against Gram-negative species [55]. In contrast, nukacin IVK45 was bacteriostatic for the Gram-negative *M*. *catarrhalis*, bactericidal for the Gram-positive *M*. *luteus*, but inactive against *S*. *aureus*. Nukacin is proposed to target the peptidoglycan precursor lipid II but may have additional modes of action as shown for other lantibiotics [56].

Our study should stimulate more extensive research on the ecological roles of bacteriocins and other fitness traits in human microbiomes. It will be particularly interesting to see how frequent and diverse bacteriocin production may be in more complex microbiota such as those of the human ileum and colon. Among oral *Streptococcus* species only 16% have been found to produce bacteriocins [57], mainly active against closely related *Streptococcus pneumoniae*, suggesting that bacteriocins may have a less important ecological role in upper gastrointestinal compared to upper respiratory microbiomes. Bacteriocins, acting against *S*. *aureus* or other endogenous facultative pathogens, and the bacteriocin-producing strains may become attractive agents for future selective decolonization strategies. The nukacin-related lantibiotic mersacidin has been successfully used to completely eradicate methicillin-resistant *S*. *aureus* (MRSA) from the nasal epithelium in a mouse model of rhinitis [58] suggesting that sophisticated bacteriocin-producing ‘probiotics’ could become valuable new agents for prevention of opportunistic infections in immune-compromised at-risk patients.

## Materials and Methods

### Bacterial strains and growth conditions

*C*. *accolens*, *C*. *pseudodiphtheriticum*, *D*. *pigrum* and *R*. *mucilaginosa* were a kind gift from the Medical Microbiology Department of Münster. *M*. *catarrhalis*, *P*. *acnes*, *and H*. *influenzae* were provided by the microbial diagnostics facility of University Hospital Tübingen. *S*. *pyogenes* BK2192, *S*. *aureus* Newman, *S*. *aureus* Newman Δ*dltA* and *S*. *epidermidis* 1457 are frequently used laboratory strains. Additionally, a nasal isolate of *M*. *luteus* was used. Tryptic soy broth (TSB) and tryptic soy agar (TSA) were used to cultivate *S*. *aureus*, *S*. *epidermidis*, *M*. *luteus*, *S*. *pyogenes*, *R*. *mucilaginosa*, *M*. *catarrhalis*, *P*. *acnes*, and *C*. *pseudodiphtheriticum*. Brain heart infusion (BHI) agar and broth were used for *C*. *accolens*. *Staphylococcus* isolates from nasal swabs from 37 healthy volunteers (IVK strains) have been described in a recent study [12]. In short, the nasal swabs were resuspended in 1 ml PBS. 100 μl of 10^−2^ and 10^−3^ dilutions were plated on blood agar plates, and single colonies were analyzed by MALDI-TOF (Matrix Assisted Laser Desorption/Ionisation-Time of Flight) for species determination.

### Ethics statement

The nasal sample collection procedure was approved by the clinical ethics committee of the University of Tuebingen (No. 109/2009 BO2) and informed written consent was obtained from all volunteers. Nasal swabs were taken exclusively from healthy adults.

### Analysis of antimicrobial activities

The nasal *Staphylococcus* isolates were stamped on TSA agar plates inoculated with the relevant test strain and incubated at 37°C. In order to prepare *S*. *aureus* and *S*. *epidermidis* test plates, autoclaved tryptic soy agar (TSA) was cooled to 45°C and inoculated with the test strains to an OD_600_ = 0.001. After mixing the medium with the cells, plates of 20 ml volume were poured. For *M*. *luteus* plates bacteria were inoculated at an OD_600_ of 0.01. For all other strains, OD_600_ = 0.1 was used.

### DNA manipulation and sequencing

For DNA manipulation standard procedures were used [59]. All restriction enzymes and High-fidelity polymerase (used for all PCRs) were obtained from Fermentas GmbH, Germany. Primers used for PCR or DNA sequencing were obtained from Eurofins MWG Operon, Germany (Table 2). DNA sequencing was done by GATC Biotech AG, Germany and Lasergene software (DNAStar, Inc., USA) was used for sequence analysis. Preparation of electro-competent *Staphylococcus* cells and electroporation was performed as described elsewhere [60].

**Table 2:** Primers used in this study.

Primer	Sequence (restriction sites underlined)	Application
Tn917 up	CTGCAATAACCGTTACCTGTTTGTGCC	sequencing of Tn917 insertion site
Ptn2 down	GGCCTTGAAACATTGGTTTAGTGGG	sequencing of Tn917 insertion site
Nukacin KO up1	GATTAAAAGAATTCAAGAGAGTTACG	construction of deletion vector pBASE6-ΔnukA
Nukacin KO up2	TTATGGTACCCCCTTTAAATTTATATATG	construction of deletion vector pBASE6-ΔnukA
Nukacin KO down1	TAAAAGCGCGCAAGGAATGCTATATCAATG	construction of deletion vector pBASE6-ΔnukA
Nukacin KO down2	GTGCGCAGTCGACTTACATAGTGGTACG	construction of deletion vector pBASE6-ΔnukA
compl.–promotor	ATTCTGCAGTAAATTTAAAGGGGGTATTATAATGG	construction of complementation vector pRB474-nukA
compl. Down	TATAGAGCTCCTTGCATGATTTTATCCAC	construction of complementation vector pRB474-nukA
Spa3-F	ATAGCGTGATTTTGCGGTT	amplification of *spa* [62]
Spa3-R	CTAAATATAAATAATGTTGTCACTTGGA	amplification of *spa* [62]

### *Spa*-typing and MLST analysis

The spa-types of all *S*. *aureus* IVK strains except two have been published recently [61]. Since the two *S*. *aureus* isolates characterized here were non spa-typeable by the common method, we amplified the whole *spa* gene with primers spa3-F and spa3-R (Table 2) [62] The PCR products were subsequently sequenced and the repeat region was analyzed using Ridom StaphType 1.5.21 software [63].

MLST of the seven house-keeping genes for *S*. *epidermidis* was performed with the primers listed on the MLST web site (http://pubmlst.org/sepidermidis/) and sequences were compared with the MLST database.

### Transposon mutagenesis

The transposon mutagenesis procedure was performed as described earlier [64]. Briefly, *S*. *epidermidis* IVK45 harboring the transposon vector pTV1ts [65] was grown in TSB containing 5 μg/ml erythromycin (Em) and 10 μg/ml chloramphenicol at 30°C overnight. The culture was then diluted in TSB containing 2.5 μg/ml Em and cultivated overnight at 42°C. After two further cycles of cultivation at 42°C with Em 2.5 μg/ml the cells were spread on TSA plates containing Em 2.5 μg/ml. Erythromycin-resistant and chloramphenicol-sensitive mutants were screened for their ability to inhibit *M*. *luteus* growth. Among 100 mutants, 10 did not inhibit the test strain *M*. *luteus*. To determine the exact insertion site, the isolated plasmid DNA was sequenced with primers Tn917 up and Ptn2 down, which read upstream and downstream of the transposon.

### Construction and complementation of a nukacin IVK45-deficient mutant

The gene for the nukacin IVK45 precursor peptide *nukA* was exchanged for an erythromycin resistance cassette. The flanking regions of *nukA* were amplified with primers Nukacin KO up1, Nukacin KO up2 (F1) and Nukacin KO down1, Nukacin down2 (F2). After digestion of F1 with EcoRI/Acc65I and F2 with BamHI/SalI, the two restricted flanking region PCR products F1 and F2 were subsequently ligated into the EcoRI/Acc65I and BglII/SalI digested vector pBase6-erm/lox2. The resulting plasmid pBASE6-ΔnukA was used to transform *E*. *coli* DC10B [66]. The plasmid was subsequently isolated and transferred to *S*. *epidermidis* IVK45, where the homologous recombination process resulted in *nukA*-deficient mutants [67], which were confirmed by PCR analysis.

For complementation, the shuttle vector pRB474 [68] was used. With primers compl.-promotor and compl. down, *nukA* was amplified. The resulting PCR product was digested with SalI/PstI and ligated into SalI/PstI-restricted pRB474. The resulting complementation vector pRB474-*nukA* was used to transform *E*. *coli* DC10B. After plasmid isolation, the vector was directly transferred to *S*. *epidermidis* IVK45 Δ*nukA* via electroporation.

### Antimicrobial activity assay for nukacin IVK45

Bioactivity was assessed by agar diffusion assays against the sensitive indicator strain *M*. *luteus*. To this end, TSA was inoculated with an overnight culture of *M*. *luteus* to a final OD_600_ of 0.01. After solidification of the agar, 10 μl of an overnight culture were put on the agar plate and incubated at 37°C. After incubation overnight, inhibitions zones were measured.

### Nukacin activity in liquid medium

Fresh TSB Medium was supplemented with sterile filtered supernatant of IVK45 wild type or the nukacin-deficient mutant, or spent TSB medium of the cultures was used directly for monitoring growth of the test strains in a photometer. The cultures were inoculated with *M*. *luteus* or *M*. *catarrhalis* (to OD_600_ = 0.01) and colony-forming units per ml were determined for several time points. *C*. *accolens* only grew in liquid BHI medium in the presence of 0.2% Tween 80. The amount of conditioned culture filtrates required for optimal growth and inhibition of potential competitors were different for the test strains corresponding to 20%-40% (*M*. *luteus*), 50%-100% (*C*. *accolens*), and 100% and enriched activity (*M*. *catarrhalis*). For the 4-fold enrichment of nukacin IVK45 activity the 100% ethanol XAD-1180 eluate, described in the nukacin IVK45 purification section, was dried and resuspended in TSB. Subsequently, the dilution factor was determined to obtain the same inhibitory activity as with the corresponding 100% culture supernatant. Experiments were performed with four-fold amount of the determined concentration.

### Co-cultivation experiments

*S*. *epidermidis* IVK45 wild type, *S*. *epidermidis* IVK45 Δ*nukA* and *M*. *catarrhalis* were grown in TSB overnight at 37°C under continuous shaking. These strains were then adjusted to 1 x 10^7^ c.f.u./ml in 1 x PBS. For the starting conditions each of the *S*. *epidermidis* strains was mixed with *M*. *catarrhalis* at a ratio of 3:1. 20 μl of these mixtures were spotted on TSB agar and incubated at 37°C. Samples were taken at 0 and 48 hours by scraping cells from the agar plates, which then were suspended in 1 x PBS. Serial dilutions of these samples were plated on TSB agar. After overnight incubation at 37°C colony counts were determined, and the bacterial ratios of *S*. *epidermidis* and *M*. *catarrhalis* were calculated. The individual strains could be distinguished by size and color of their colonies.

### Nukacin IVK45 purification

XAD-1180 resin, cation exchange (SP Sepharose FF column), and size exclusion (Sephadex LH-20 column) chromatography were used to purify nukacin IVK45 from liquid cultures. Cell-free culture supernatant was incubated with 1/10 volume of XAD-1180 adsorber resin (Acros Organics) and incubated for 2 h under constant agitation. Subsequently XAD-1180 was washed twice with 2 x bed volumes (BV) 60% ethanol. For elution 2 x BV of 100% ethanol were applied. Subsequently, ethanol was removed using a rotary evaporator. The resulting extracts were dissolved in 25 mM sodium phosphate buffer pH 7.2 (binding buffer). The sample was injected into a fast protein liquid chromatography (FPLC) system (ÄKTA Purifier) using a 10-ml SP Sepharose FF (strong) cation exchanger column (GE Healthcare) equilibrated with the same buffer at a flow rate of 1 ml/min. Then the column was washed with a 10-fold column volume of the binding buffer before nukacin IVK45 was eluted using the following step gradient: 1.5 M NaCl in 25 mM sodium phosphate buffer at pH 7.2 was used as elution buffer (Buffer B); 25 min 10% buffer B, 35 min 13% buffer B, 20 ml 100% buffer B; nukacin IVK45 eluted immediately after the gradient reaches more than 13% of buffer B. All fractions were tested for inhibitory activity using *M*. *luteus* as the indicator strain.

The active SP Sepharose fraction of the wild type and the corresponding fraction of the deletion mutant were freeze dried, dissolved in 100% methanol, and subsequently separated via a 60-ml Sephadex LH-20 size exclusion column (GE healthcare) with methanol as running solvent. In the active fractions nukacin IVK45 was detected by High Pressure Liquid chromatography–Mass Spectrometry (HPLC-MS).

### Nukacin analysis by HPLC electrospray Ionization mass spectrometry (ESI-MS)

The methanolic fractions obtained from the size exclusion chromatography were analyzed by a HPLC-LC/MSD Ultra Trap System XCT 6330 (Agilent Technologies). For the HPLC measurements, a C18 Nucleosil 100 column (100 x 2 mm ID, 3 μm) and a pre-column (100 x 2 mm ID) (Dr. Maisch) were used. Solvent A (0.1% formic acid) and solvent B (0.06% formic acid in acetonitrile) were used as mobile phase applying the following gradient (t_0_ = 10% B, t_15_ = t_17_ = 100% B, post-time 5 min. 10% B; flow rate 400 μl/min; injection volume 2.5 μl). UV signals were detected at: 220 nm (10 nm band width), 260 nm (20 nm), 280 nm (20 nm), 360 nm (20 nm), and 435 nm (40 nm). Data analysis was accomplished with the Agilent LC/MSD software ChemStation Rev. B.01.03, Agilent. The following parameters were used for the MS detection: ionisation: ESI (alternating positive and negative ionization) in ultra-scan mode; capillary voltage: 3.5 kV target mass: m/z 1500. Data analyses were performed with the software 6300 Series Trap Control Version 6.1.

## Supporting Information

S1 Fig Antimicrobial activities of IVK strains. Examples for the antimicrobial activity assay with IVK strains 1–96 stamped on agar plates with *M*. *luteus* as indicator strain.
(DOCX)

S2 Fig Induction of antimicrobial activity by selected IVK strains. No stressors added (A and C), 0.01% H_2_O_2_ (B), iron limitation (D). *M*. *luteus* was used as test strain.
(DOCX)

S3 Fig Alignment of prepeptide sequence of nukacin IVK 45 with other lantibiotics and hypothetical peptides from various bacterial genomes. Matching amino acids are highlighted; the vertical arrow indicates the processing site. Sequences are labeled as follows: The *S*. *epidermidis*, *S*. *warneri*, and *S*. *hominis* sequences correspond to nukacin variants. The other peptides are encoded in genomes of the indicated species. Strain abbreviations: NUK IVK45_*Staphylococcus epidermidis* IVK45; NUK KQU-131_*Staphylococcus hominis* KQU-131; NUK ISK-1_*Staphylococcus warneri* ISK-1; SAG 14609 *Streptooccus agalactiae* 14609; NUK Ss_*Streptococcus suis*; NUK Ss2_*Streptococcus suis*; BUTYV_*Butyriovibrio fibrisolvens* OR79; KOC BAC_*Kocuria varians*; LAC 481_*Lactococcus lactis*; LAC J46_*Lactococcus lactis*; LAC 481-l_oral *Actinomyces* sp.; RUM C_*Streptococcus equi* subsp. *ruminatorum* C; NUK SAL K12_*Streptococcus salivarius* K12; NUK SAL_*Streptococcus salivarius*; SAL G32_*Streptococcus salivarius* G32. Color definition: yellow: highest identity in the leader peptide; green: highest identity in the mature peptide (variation in maximum one amino acid); lilac: high identity in the mature peptide (variation in maximum two amino acids); light red: amino acids characteristic for nukacin peptides; light blue: highly conserved amino acids differing from nukacin peptides.
(DOCX)

S4 Fig HPLC/ESI-MS analysis of nukacin IVK 45. HPLC UV/Vis elution profile of IVK 45 wild type (A) or nukacin IVK 45-deficient mutant (B) extract. C: deconvoluted spectrum generated with MagTran 1.03.
(DOCX)

S1 Table Multilocus sequence typing (MLST) of *S*. *epidermidis* isolates used in this study. (DOCX)

S2 Table Spa typing of *S*. *aureus* isolates used in this study. (DOCX)
