# Supplementary figures and images for: High Frequency and Diversity of Antimicrobial Activities Produced by Nasal Staphylococcus Strains against Bacterial Competitors
Source: PLoS Pathog. 2016 Aug 4;12(8):e1005812. doi: 10.1371/journal.ppat.1005812 (PMC4973975; doi:10.1371/journal.ppat.1005812)

**
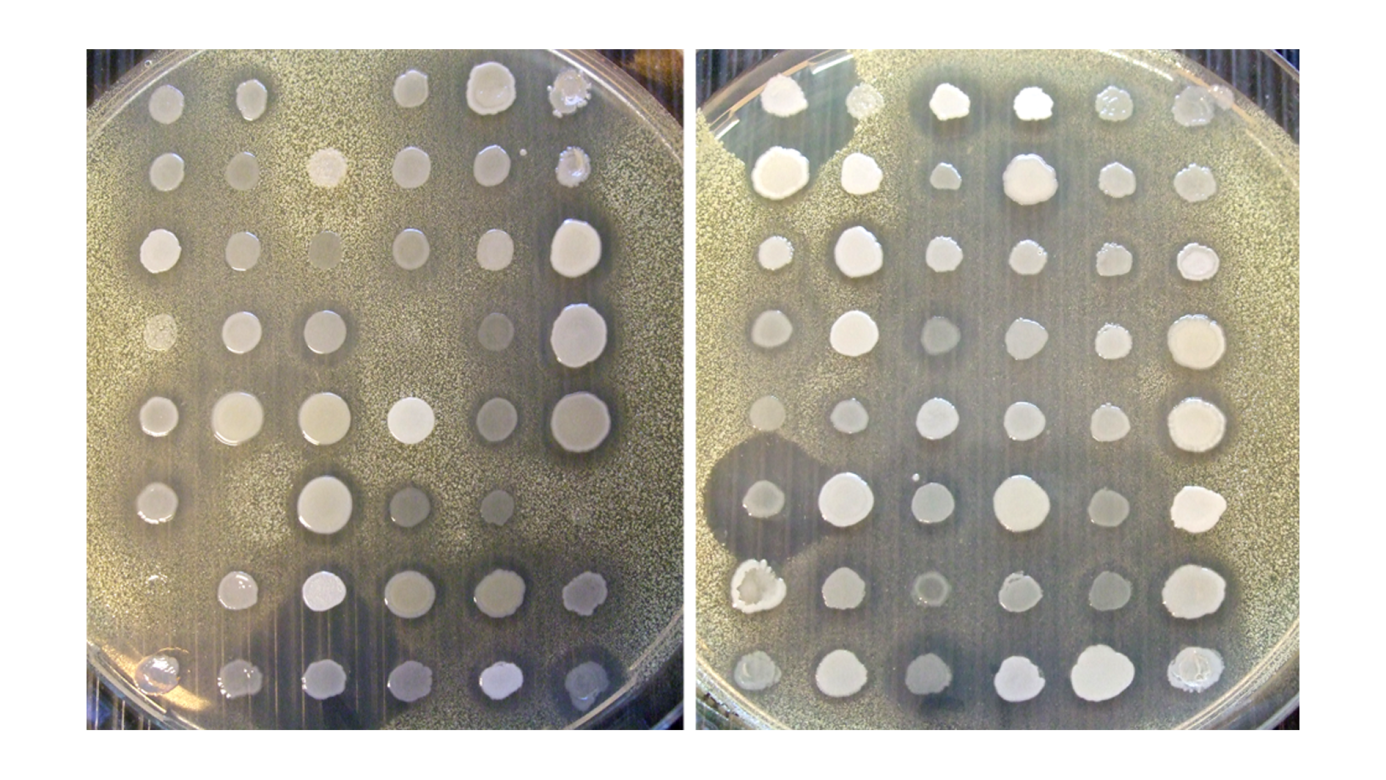
**

Supplement: S1 Fig — Examples for the antimicrobial activity assay with IVK strains 1–96 stamped on agar plates with M. luteus as indicator strain. (DOCX) [file ppat.1005812.s001.docx]

**
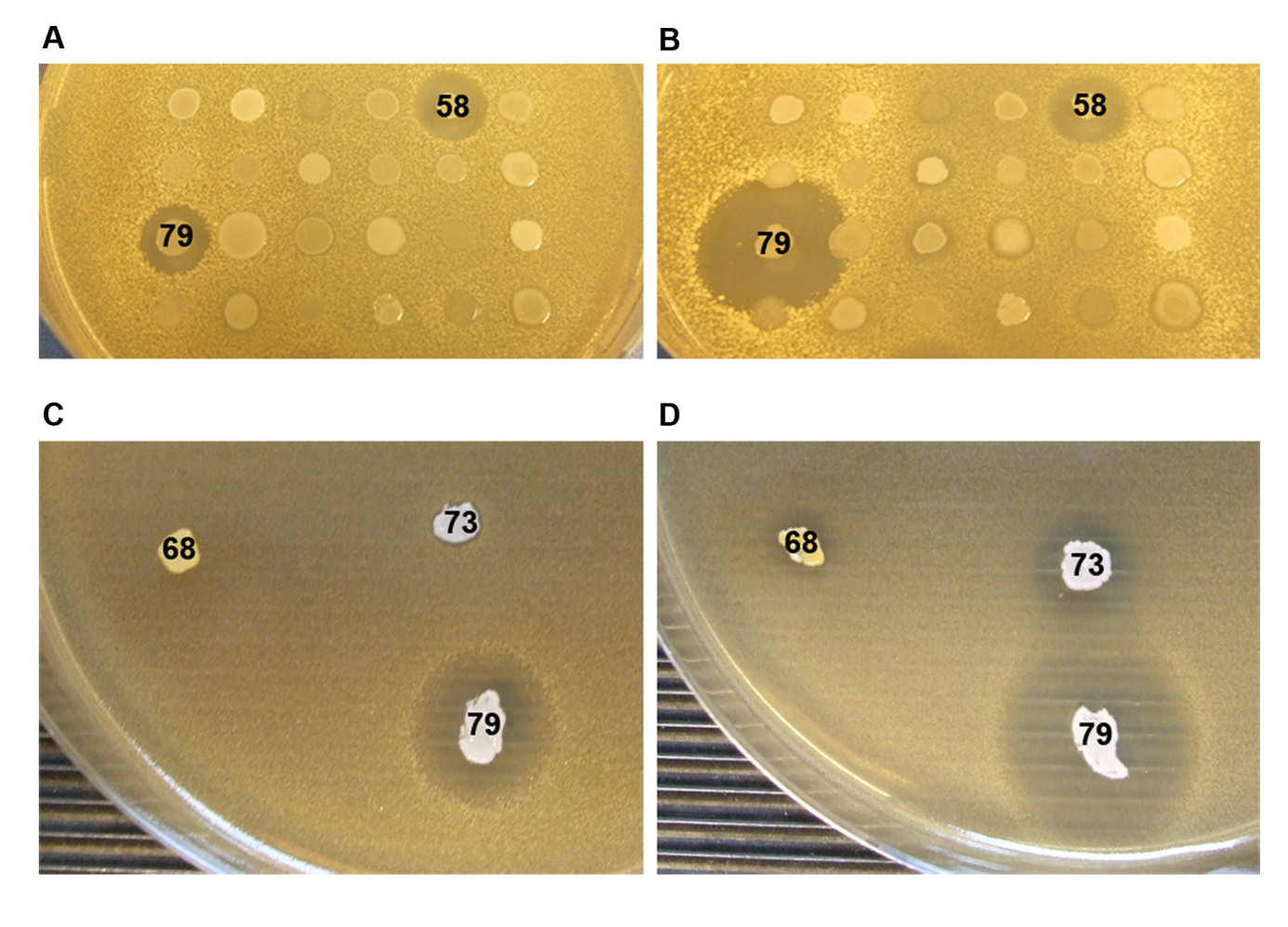
**

Supplement: S2 Fig — No stressors added (A and C), 0.01% H2O2 (B), iron limitation (D). M. luteus was used as test strain. (DOCX) [file ppat.1005812.s002.docx]

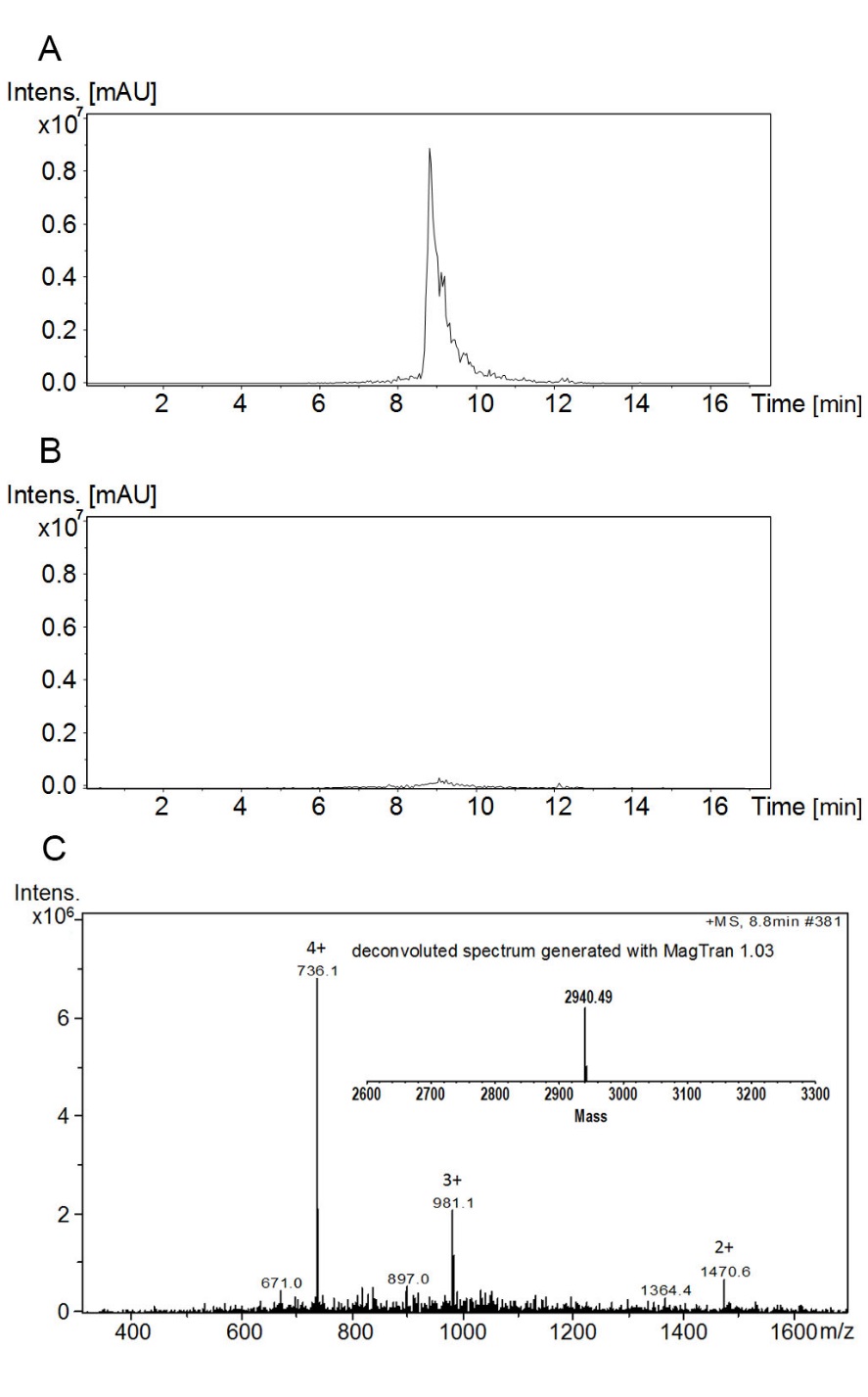

Supplement: S4 Fig — HPLC UV/Vis elution profile of IVK 45 wild type (A) or nukacin IVK 45-deficient mutant (B) extract. C: deconvoluted spectrum generated with MagTran 1.03. (DOCX) [file ppat.1005812.s004.docx]
