# Supplementary material for: High Frequency and Diversity of Antimicrobial Activities Produced by Nasal Staphylococcus Strains against Bacterial Competitors
Source: PLoS Pathog. 2016 Aug 4;12(8):e1005812. doi: 10.1371/journal.ppat.1005812 (PMC4973975; doi:10.1371/journal.ppat.1005812)
Supplement: S1 Table — (DOCX) [file ppat.1005812.s005.docx]

**Supplementary Table S1: Multilocus sequence typing (MLST) of *S. epidermidis* isolates used in this study:**

| ***S. epidermidis*** | ***arcC*** | ***aroE*** | ***gtr*** | ***mutS*** | ***pyrR*** | ***tpiA*** | ***yqiL*** | **sequence** **type (ST)** | **inhibitory pattern** |
| --- | --- | --- | --- | --- | --- | --- | --- | --- | --- |
| strain ID | allele no. | allele no. | allele no. | allele no. | allele no. | allele no. | allele no. |  |  |
| IVK14 | 1 | 5 | 2 | 6 | 2 | 1 | 6 | **73** | 4 |
| IVK1 | 16 | 1 | 2 | 1 | 2 | 1 | 1 | **184** | 5 |
| IVK8 | 1 | 5 | 2 | 6 | 2 | 1 | 6 | **73** | 5 |
| IVK23 | 1 | 1 | 2 | 6 | 2 | 16 | 1 | **218** | 5 |
| IVK59 | 1 | 2 | 2 | 2 | 2 | 1 | 3 | **297** | 5 |
| IVK74 | 2 | 1 | 1 | 1 | 2 | 1 | 1 | **59** | 6 |
| IVK10 | 2 | 1 | 1 | 1 | 1 | 2 | 1 | **466** | 7 |
| IVK11 | 1 | 1 | 1 | 2 | 5 | 1 | 1 | **190** | 7 |
| IVK12 | 2 | 1 | 1 | 1 | 2 | 1 | 1 | **59** | 7 |
| IVK15 | 1 | 5 | 2 | 6 | new (2 like) | 1 | 16 | new ST | 7 |
| IVK16 | 1 | 1 | 2 | 6 | 2 | 16 | 1 | **218** | 7 |
| IVK20 | 1 | 1 | 2 | 6 | 2 | 16 | 1 | **218** | 7 |
| IVK34 | 2 | 1 | 1 | 1 | 2 | 1 | 1 | **59** | 7 |
| IVK35 | 1 | 5 | 2 | 6 | 2 | 1 | 16 | new ST | 7 |
| IVK48 | 50 | new (3 like) | new (5 like) | 5 | 2 | 1 | 45 | new ST | 7 |
| IVK53 | 1 | 1 | 2 | 6 | 2 | 16 | 1 | **218** | 7 |
| IVK65 | 1 | 1 | new (2 like) | new (1 like) | 2 | 1 | 7 | new ST | 7 |
| IVK77 | 1 | 1 | 9 | 5 | 2 | 1 | 1 | **284** | 7 |
| IVK89 | 2 | 1 | 1 | 1 | 2 | 1 | 1 | **59** | 7 |
