# Supplementary material for: High Frequency and Diversity of Antimicrobial Activities Produced by Nasal Staphylococcus Strains against Bacterial Competitors
Source: PLoS Pathog. 2016 Aug 4;12(8):e1005812. doi: 10.1371/journal.ppat.1005812 (PMC4973975; doi:10.1371/journal.ppat.1005812)
Supplement: S2 Table — (DOCX) [file ppat.1005812.s006.docx]

| **Isolate** | **source** | **spa-type** | **MLST** |  |
| --- | --- | --- | --- | --- |
| *S. aureus* IVK 5 | Wild type, nasal isolate | **t223** | **ST-22** | [61] |
| *S. aureus* IVK 18 | Wild type, nasal isolate | **t645** |  | [61] |
| *S. aureus* IVK 24 | Wild type, nasal isolate | **t4309** |  | [61] |
| *S. aureus* IVK 26 | Wild type, nasal isolate | **t1487** |  | [61] |
| *S. aureus* IVK 30 | Wild type, nasal isolate | **t084** | **ST-15/18** | This study |
| *S. aureus* IVK 33 | Wild type, nasal isolate | **t177** | **ST-3** | [61] |
| *S. aureus* IVK 40 | Wild type, nasal isolate | **t012** | **ST-30** | [61] |
| *S. aureus* IVK 41 | Wild type, nasal isolate | **t012** | **ST-30** | This study |
| *S. aureus* IVK 55 | Wild type, nasal isolate | **t442** | **ST-487** | [61] |
| *S. aureus* IVK 56 | Wild type, nasal isolate | non-typeable |  | This study |
| *S. aureus* IVK 58 | Wild type, nasal isolate | **t136** |  | [61] |
| *S. aureus* IVK 62 | Wild type, nasal isolate | **t077** |  | [61] |
| *S. aureus* IVK 72 | Wild type, nasal isolate | **t190** | **ST-8** | [61] |
| *S. aureus* IVK 78 | Wild type, nasal isolate | **t668** |  | [61] |
| *S. aureus* IVK 80 | Wild type, nasal isolate | **t056** | **ST-101** | [61] |
| *S. aureus* IVK 82 | Wild type, nasal isolate | **t122** |  | [61] |
| *S. aureus* IVK 85 | Wild type, nasal isolate | **t078** | **ST-26** | [61] |
| *S. aureus* IVK 90 | Wild type, nasal isolate | **t015** | **ST-45** | [61] |
| *S. aureus* IVK 95 | Wild type, nasal isolate | **t1510** |  | [61] |

**Supplementary Table S2: Spa typing of *S. aureus* isolates used in this study**
