# Supplementary material for: High Frequency and Diversity of Antimicrobial Activities Produced by Nasal Staphylococcus Strains against Bacterial Competitors
Source: PLoS Pathog. 2016 Aug 4;12(8):e1005812. doi: 10.1371/journal.ppat.1005812 (PMC4973975; doi:10.1371/journal.ppat.1005812)
Supplement: Supplementary file 4 [file ppat.1005812.s003.docx]

```
                        Nukacin cleavage site
                ---------+---------+---------+---------+---------+---------+-----
                         10        20        30    ↓   40        50        60
                ---------+---------+---------+---------+---------+---------+-----
NUK IVK45       MENFKVIE------DIEVSNLLEEIQEDELNEVLGAKKKS--GAVPTVSHDCHMNSWQFIFTCCG
NUK KQU-131     MENSKIMK------DIEVANLLEEVQEDELNEVLGAKKKS--GVIPTVSHDCHMNTFQFMFTCCS
NUK ISK-1       MENSKVMK------DIEVANLLEEVQEDELNEVLGAKKKS--GVIPTVSHDCHMNSFQFVFTCCS
SAG 14609 hyp   MK-------NTNIIDIEATNALQELSFEELDTIIGAKKKGNDGAIPTISHDCHMNSWQFIFTCCS
NUK Ss          MK-------K----DIELMSTIQEVSLEELDQIIGA---GKNGVFKTISHECHMNSWQFLFTCCS
NUK2 Ss         MK-------D----NYELMNTIQEVSLEELDQIIGA---GKNGVFKTISHECHMNSWQFLFTCCS
BUTYV           MNK-----------ELNALT--NPIDEKELEQILGG----GNGVIKTISHECHMNTWQFIFTCCS
KOC BAC         MTN--------------AFQALDEVTDAELDAILGG----GSGVIPTISHECHMNSFQFVFTCCS
LAC 481         M------K------EQNSFNLLQEVTESELDLILGA--KGGSGVIHTISHECNMNSWQFVFTCCS
LAC J46         M------K------EQNSFNLLQEVTESELDLILGA--KGGSGVIHTISHEVIYNSWNFVFTCCS
LAC 481-l       MSNINM----------QAAAALDELSDAELDQVLGA----GNGVYYTLSHECHMNSWQFIFTCCS
RUM C           MK-------D----NYELMNTIQEVSLEELDQIIGA---GKNGAIKTISHECHMNSWQFLFTCCS
NUK SAL K12     M-----AKQQMNLVEIEAMNSLQELTLEELDNVLGA----GGGVIQTISHECRMNSWQFLFTCCS
NUK SAL         MRYFQMAKQQMNLVEIEAMNSLQELTLEELDNVLGA----GGGVIQTISHECRMNSWQFLFTCCS
SAL G32         MK-------K----DV-VIESIKEVSLEELDQIIGA----GNGVFKTISHECHLNTWAFLATCCS
```
